# Observation of a moderate major Baltic Sea inflow in December 2023

**DOI:** 10.1038/s41598-024-67328-8

**Published:** 2024-07-17

**Authors:** Kaveh Purkiani, Kerstin Jochumsen, Jens-Georg Fischer

**Affiliations:** Federal Maritime and Hydrographic Agency of Germany, Hamburg, Germany

**Keywords:** Physical oceanography, Climate-change impacts

## Abstract

Just before Christmas 2023, the low-pressure system storm “Zoltan” struck Germany, resulting in widespread damage and two consecutive large storm surges on the North Sea coast in the night from Thursday 21 December 2023 to Friday 22 December 2023. Storm Zoltan brought heavy rainfall, accompanied by thunderstorms and winds ranging between 90 and 115 $\mathrm {km\,h^{-1}}$, with gusts reaching up to 140 $\mathrm {km\,h^{-1}}$ along the coast which caused severe damage, particularly in northern Germany. Characteristics of the inflowing water at the Fehmarn Belt buoy (FEB), Darss Sill station (DAR) and the Arkona Basin buoy (ARK), including salinity, temperature, dissolved oxygen and ocean currents properties, were analysed to understand the impact of storm Zoltan in the western Baltic Sea. In addition to the damage along its path, following the onset of strong westerly winds associated with storm Zoltan, a large volume of water, containing saline (17–22 psu), cold (5–6 °C), and oxygen-rich 7–8 ($\,\mathrm { ml\,l^{-1}}$) water from the Kattegat and the North Sea reached into the western Baltic Sea. The sea level at Landsort Norra increased by +57 cm over a period of 14 days, from 15 December to 29 December 2023. This resulted in a total volume change of 198 $\mathrm {km^3}$ in the entire Baltic Sea, with 169 $\mathrm {km^3}$ and 29 $\mathrm {km^3}$ provided via the Belt Sea and the Sound Sea, respectively. Observations at the DAR indicated a significant inflow between 19 December 2023 and 1 January 2024 with salinity above 13 psu, temperature below 5.5 °C and dissolved oxygen of about 7.5–8 $\mathrm { ml\,l^{-1}}$. While the maximum salinity of the bottom layer at the DAR was about 17 psu, the ARK exhibited significantly higher salinities, reaching up to 22 psu at the bottom layer. During the main inflow period, 75 $\mathrm {km^3}$ of highly saline water entered the western Baltic Sea. This corresponds to an average salt transport of 1.75 Gt into the western Baltic Sea (1.39 Gt from the Belt Sea and 0.36 Gt from the Sound Sea), representing more than 20% of the total annual salt import into the Baltic Sea), which places the event in the moderate range of major Baltic inflows. This event brought an amount of about 0.8 $\mathrm {10^6}$ t oxygen into the Baltic Sea. This was the strongest inflow into the Baltic Sea since 2016.

## Introduction

**Figure 1:**
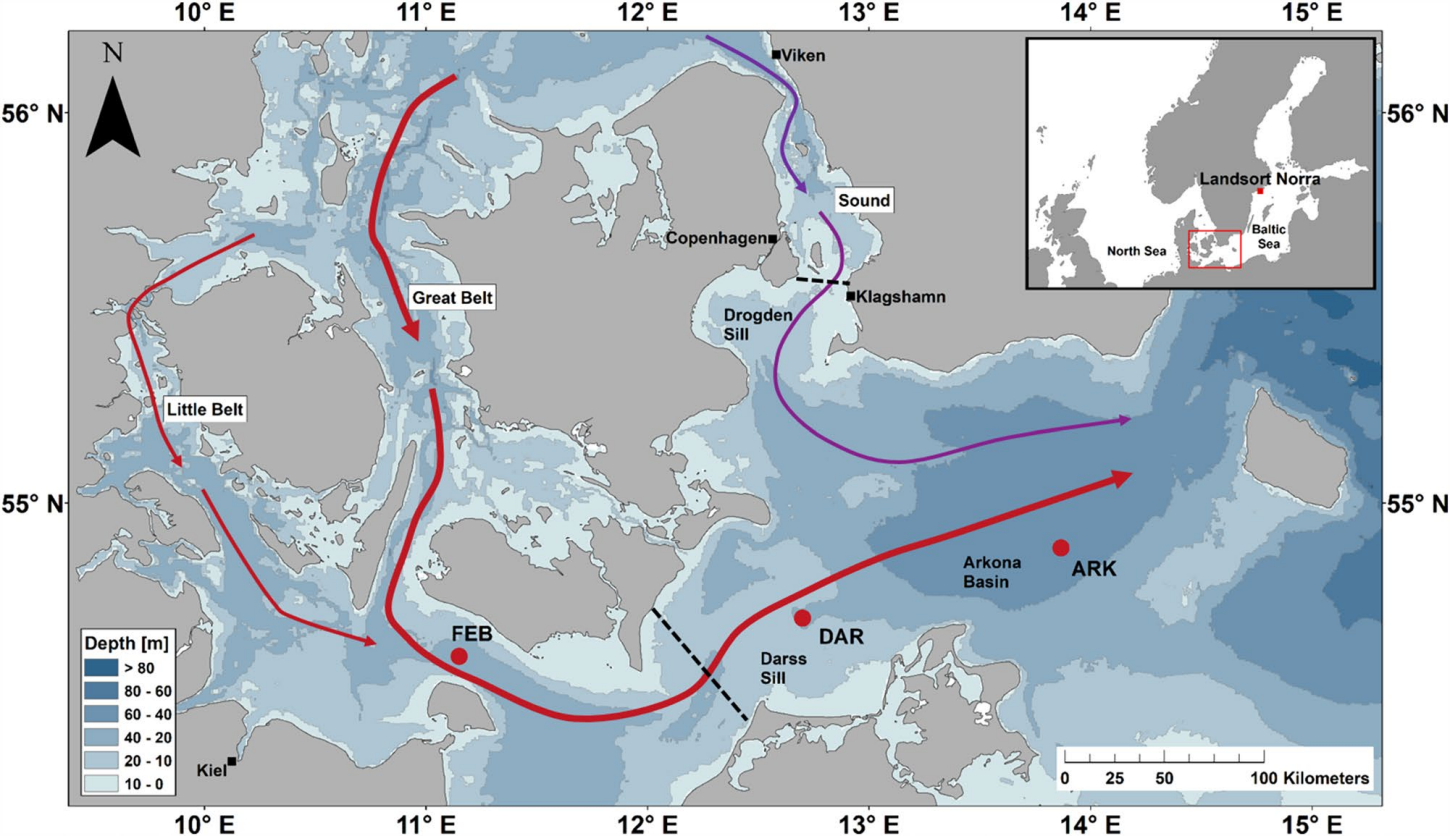
Bathymetric map of the southwestern Baltic Sea, along with locations where measurements were taken as red dots ( Fehmarn Belt buoy-FEB; Darss Sill station-DAR; Arkona Basin buoy-ARK). The Baltic Sea connects to the Kattegat and North Sea via shallow straits: the Belt Sea (Little and Great Belt) and the Sound. Colored arrows on the map depict the separate pathways of the inflowing salty water through these connections. Along with sills (Darss and Drogden) crucial for water exchange, depicted by dashed lines, the map also shows the Arkona Basin, one of the Baltic Sea’s first deeper basins along the pathway of the inflowing water, where depths of up to 48 meters are reached. Black squares on the map represent tide gauge stations located at Viken, Klagshamn, and Landsort Norra.

Storms in the North Sea play an essential role in shaping the dynamic environment of the region and are often associated with coastal flooding and inundation, disruptions in transportation networks, challenges in energy generation, and fatalities^1–4^. While these natural phenomena can be destructive, they also bring significant benefits by the generation of oceanic volume transport, i.e. inflow events, from the North Sea into the Baltic Sea. The connection of these shelf seas is no wide passage, but formed by several pathways along channels between islands, known as the Danish Straits (Fig. 1). The depth at Dogden Sill is only 7 m, thus limiting the water exchange. The flow through the Belt Sea is less restricted, but then has to pass over Darss Sill with a depth of 18 m^5^. North of the straits in the shallow Kattegat the transition zone between brackish waters from the Baltic Sea and oceanic waters from the North Sea is found^6^. Due to the hydrographic conditions in the Baltic Sea, the ventilation of its eastern basins mainly depends on lateral inflows^5,7^, as strong permanent water stratification prevents surface-bottom water exchange^8^. The inflows are therefore essential for oxygenating and maintaining the ecosystem of the Baltic Sea^9,10^. Earlier storm events during the last decade, e.g. Christian (October 2013), Xaver (December 2013), Danli, Ev and Feliz (February to March 2014) and their impacts on the volume transport into the Baltic Sea and the oxygenation of the Bornholm Basin were addressed in previous studies^11,12^.

Just before Christmas 2023, a low-pressure system called “Zoltan” in Germany and “Pia” in Europe (hereafter Zoltan), struck Germany, resulting in widespread damage and two consecutive large storm surges on the North Sea coast in the night from 21 December 2023 to 22 December 2023 (internal report published in German^13^) . As given in the online report of the German Weather Service, storm Zoltan brought heavy rainfall, thunderstorms and winds between 90 and 115 $\mathrm {km\,h^{-1}}$, with gusts of up to 140 $\mathrm {km\,h^{-1}}$, along the coast^14^ . In addition to major rail disruptions , extreme flooding occurred in northern Germany. For instance, in Hamburg’s St. Pauli district, the sea level rose to more than 3 meters above mean high water level on 22 December 2023 . Beside causing damage along its pathway, storm Zoltan forced a large volume of water containing salty, cold, and oxygen-rich water from the North Sea and Kattegat to enter the western Baltic Sea. Such barotropic inflows induced by storms play a significant role in the salt and oxygen budgets of the Baltic Sea and the inflow caused by Zoltan is the topic of this study.

Episodic barotropic inflows of large volumes of saline and oxygenated water, the so-called Major Baltic Sea Inflows^15,16^ (MBI), are the most important mechanisms of central Baltic deep water ventilation. To distinguish between regular inflows and MBIs, following criteria were defined using data from the light ship “Gedser Rev”, which was approx. 50 km west of the Darss Sill^5,17–19^: (i) the bottom salinity must be $\ge$ 17 psu (ii) the salinity stratification defined as $G=1-S_s/S_b$ must be $\le$ 0.2 (where $S_s$ is the surface salinity and $S_b$ is the bottom salinity) and (iii) these conditions must be persistent for at least five consecutive days. Previous analysis have shown, that easterly winds lowering the Baltic Sea sea level and following prevailing westerly winds with strong gales are a decisive factor for the occurrence of MBIs^15^. Statistical analyses of frequencies and intensities of inflow events^5,19,20^ indicate the occurrence of weak MBIs to be regular features. However, very few strong MBIs were observed in the last decades (they are: the 1993 MBI^21–23^, the 2003 MBI^24,25^ and the 2014 MBI^26^) that could turn the deep Gotland Basin to oxic conditions. The low frequency of strong MBIs led to long lasting stagnation periods, which is now the common state of the central deep Baltic without any strong MBI since 2014^8,20^.

The strong winds of Zoltan and the stormy conditions in the North Sea during the following week induced the formation of an MBI, which had the potential to bring saline and oxygen-rich water from the North Sea and Kattegat into the Arkona and Bornholm Basins. Depending on the intensity of the inflow event, the spreading of the water may have continued into the central basins and improved the oxygen situation in the deep waters of the Gotland Basin, which suffered again from a 10-year time span of stagnation. As a consequence of the extend periods of time between MBIs, the oxygen deficiency in the deeper layers of the Baltic Sea has already reached the highest known level^27^. In this study, using hydrographic observations from three locations in the Baltic Sea (Fehmarn Belt, Darss Sill and Arkona Basin), we aimed to investigate how storm Zoltan affected the hydrodynamics of the western Baltic Sea and to quantify the inflow event.

## Results

### Wind and sea level height variations

**Figure 2:**
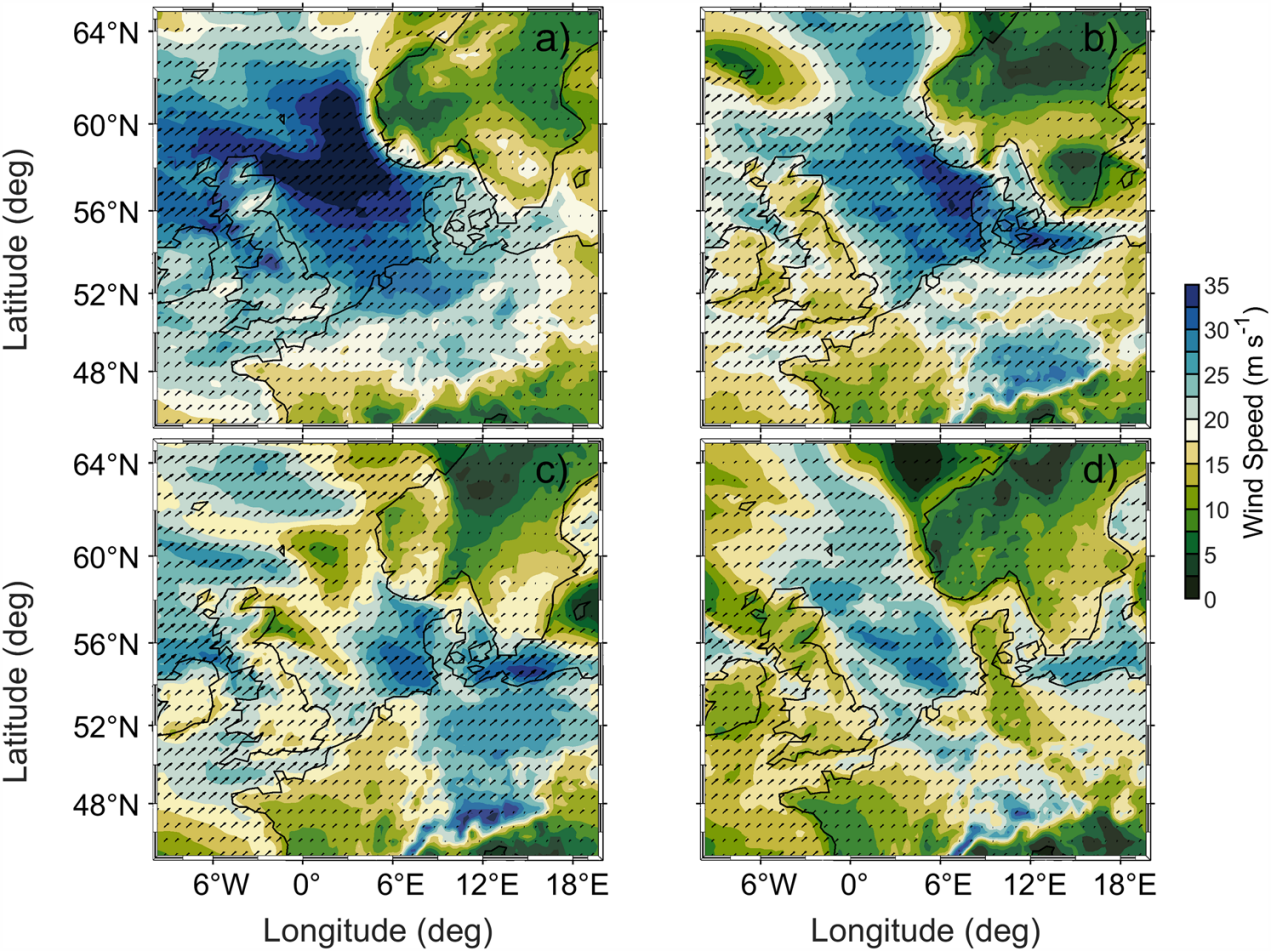
Atmospheric conditions during storm Zoltan in the North Sea and western Baltic Sea. Panels (**a**)−(**d**) show 10 m wind gust (background colour) and 10 m wind speed (black arrows) on 21 December 2023 12:00, on 21 December 2023 23:00, on 22 December 2023 09:00, and on 22 December 2023 18:00 respectively. The data were extracted from the reanalysis ERA5^32^, where they are provided in hourly resolution.

**Figure 3:**
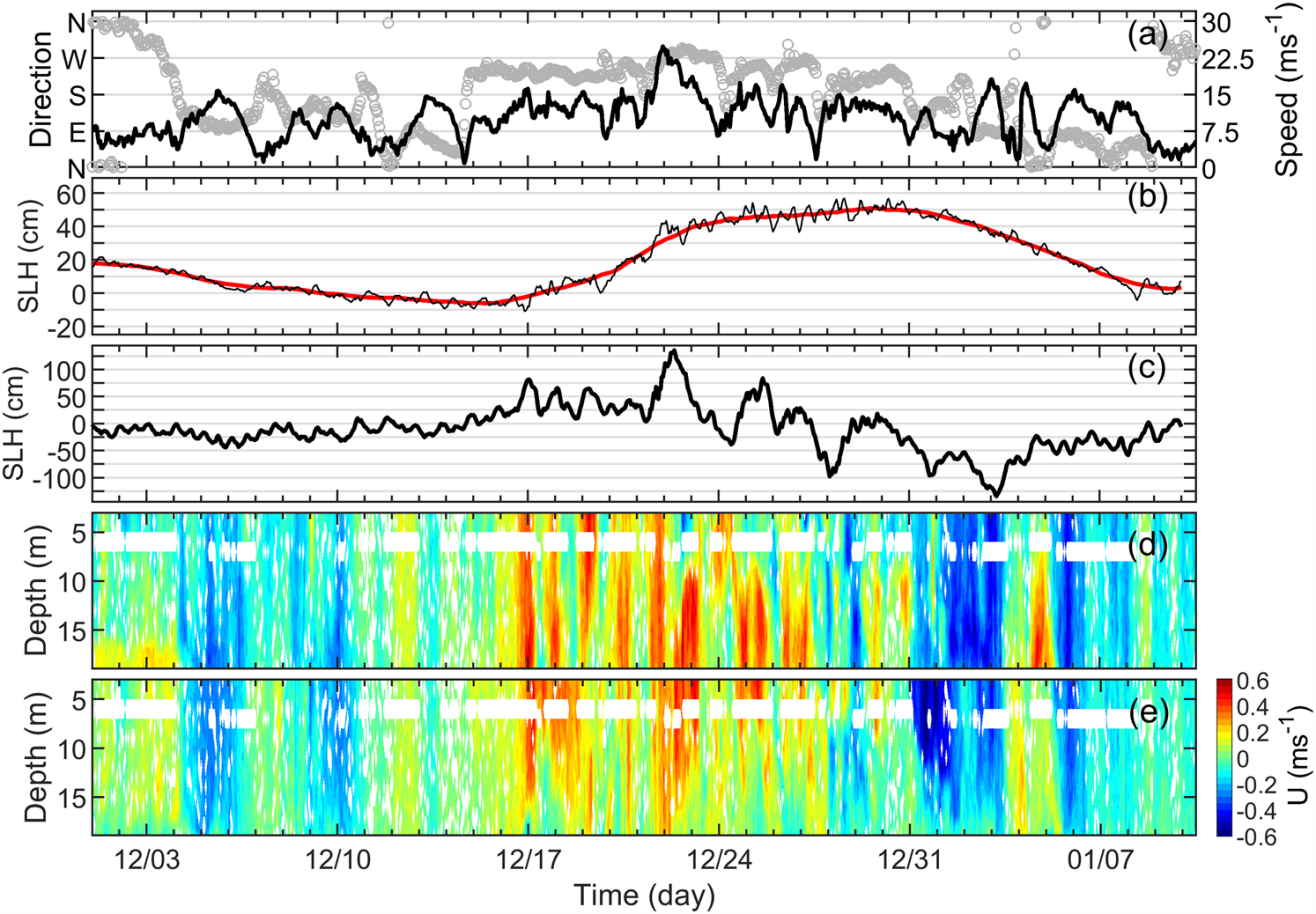
Evolution of wind, sea level height and currents during the inflow event in December 2023. (**a**) Wind speed and direction respectively in black line and grey circles between December 2023 and January 2024 from the Darss Sill station (DAR) . (**b**) Hourly sea level change (black line) at Landsort Norra during the same time in the western Gotland basin . The red thick line depicts the smoothed data using a moving average filter with a cutoff period of 3 days. (**c**) Difference in sea level between Viken and Klagshamn representing variations in sea level in the Sound Sea. (**d**) along channel and (**e**) cross channel current velocity profiles at the DAR. The u and v-components of the current velocity were rotated 35 deg.

Between 15 and 30 December 2023, a low-pressure system (968 hPa) with strong southwesterly (SW) wind direction developed in the North Sea and the Baltic Sea with wind speeds of more than 31 $\mathrm {m s^{-1}}$ in the German North Sea (Fig. 2). The wind gusts showed even higher wind speeds of up to 35 $\mathrm {m s^{-1}}$. The maximum wind speed reached the German North Sea coast on 21 December at 22:00. The strong winds had west to southwest (W-SW) direction at the Kattegat and in the western Baltic Sea (Fig. 2a–d). The maximum wind speed of 24.8 $\mathrm {ms\,^{-1}}$ was recorded at the Darss Sill Station (DAR) on 21 December at 23:00 (Fig. 3a). This was a favourable wind situation for the generation of an inflow event into the Baltic Sea, particularly following the relatively strong easterly winds which had caused an outflow in early December (Fig. 3a). After the storm had passed, a series of again relatively strong easterly winds of $18 \,\mathrm {m s^{-1}}$ were observed at the DAR from 2 to 9 January 2024.

Changes in sea level height (SLH) at the Landsort Norra station in the western Gotland Basin, which reflect the water level of the central Baltic Sea, are shown in Fig. 3b. As the wind system developed, the SLH at Landsort Norra began to rise from − 6 cm on 15 December to +51 cm on 29 December 2023. This resulted in a SLH increase of +57 cm in 14 days, implying an average SLH increase rate of 4 $\mathrm {cm\, d^{-1}}$ in the Baltic Sea for this event. Due to the very small cross-sectional area of the Sound (0.1 $\mathrm {km^2}$, 7 m sill depth), the variations in SLH were quite pronounced there, reaching up to +130 cm during this period (Fig. 3c). Compared to the MBI of December 2014, the December 2023 inflow was characterized by a shorter duration and a weaker intensity in the SLH variation. In the earlier event, a SLH variation of +103 cm was observed within 23 days^26^, resulting in an average SLH increase rate of 4.5 $\mathrm {cm\,d^{-1}}$. The inflow at DAR was observed in the velocity profile illustrated at Fig. 3d–e, where strong northeastward flow (positive v- and u-components) was recorded during the inflow period, indicating the propagation of the inflowing water into the Arkona Basin. The along channel speed of the ocean currents reached values of up to 0.6 $\mathrm {m\,s^{-1}}$ in the whole water column during that time.

### Seawater characteristics at the Darss Sill

**Figure 4:**
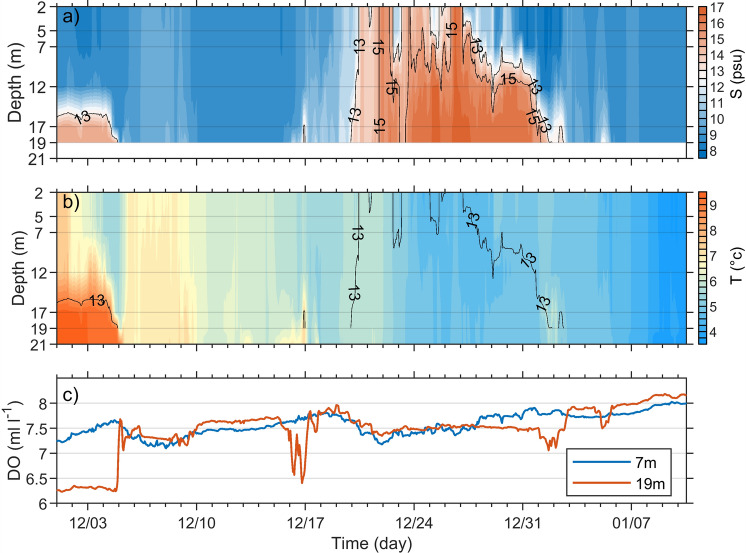
Vertical profiles of salinity (**a**) and temperature (**b**) from 1 December 2023 to 10 January 2024 at the Darss Sill station (DAR). The dissolved oxygen concentrations at 7 m and 19 m are depicted in (**c**). The isohalines of 13 and 15 psu are given in the salinity time series in (**a**) and the isohaline of 13 psu is shown again in (**b**). Hourly averages of the original higher resolution measurements were used here. The vertical axes in (**a**) and (**b**) not only indicate the depth, but also show the position of the instruments in the water column. Salinity at 21 m was not available due to a lack of measurements.

The water salinity at DAR was about 8–9 psu in the entire water column from 10 December to 16 December 2023 (Fig. 4a). As the sea level increased, the more saline water from the Kattegat and North Sea reached the DAR and the seawater salinity increased to 13 psu on 20 December 2023. The salinity continuously increased to its maximum of 16.98 psu at 19 m water depth on 25 December and the stratification remained low (Fig. 4a). Afterward (on 28 December), the surface salinity decreased to values below 10 psu, while the water column below 7 m was still filled with saline water ($S>13$ psu). The deeper layers showed a salinity of more than 15 psu for a period of about 12 days from 20 December 2023 to 2 January 2024 13:00. On 3 January 2024, the salty water disappeared from the station and the seawater salinity returned to values below 10 psu (Fig. 4a).

During the inflow period and specifically when the inflow core with $S>$ 15 psu reached the DAR, the temperature of the seawater decreased from 6 °C on 21 December to 4.9 °C on 23 December 2023 (Fig. 4b). The dissolved oxygen concentration of the inflowing water at the bottom was about 8 $\mathrm {ml\,l^{-1}}$ at the onset of the event and decreased to 7.5 $\mathrm {ml\,l^{-1}}$ during the majority of the inflow period (Fig. 4c). This is equivalent to a saturated oxygen level between 95 and 92% during the inflow period while the surface oxygen saturation showed higher values ranging from 97 to 104%. An increase of about 1.5 $\mathrm {ml\,l^{-1}}$ in oxygen concentration was evident in the bottom water, when values were compared to the conditions in early December (Fig. 4c).

### Inflow characteristics at the Arkona Basin

**Figure 5:**
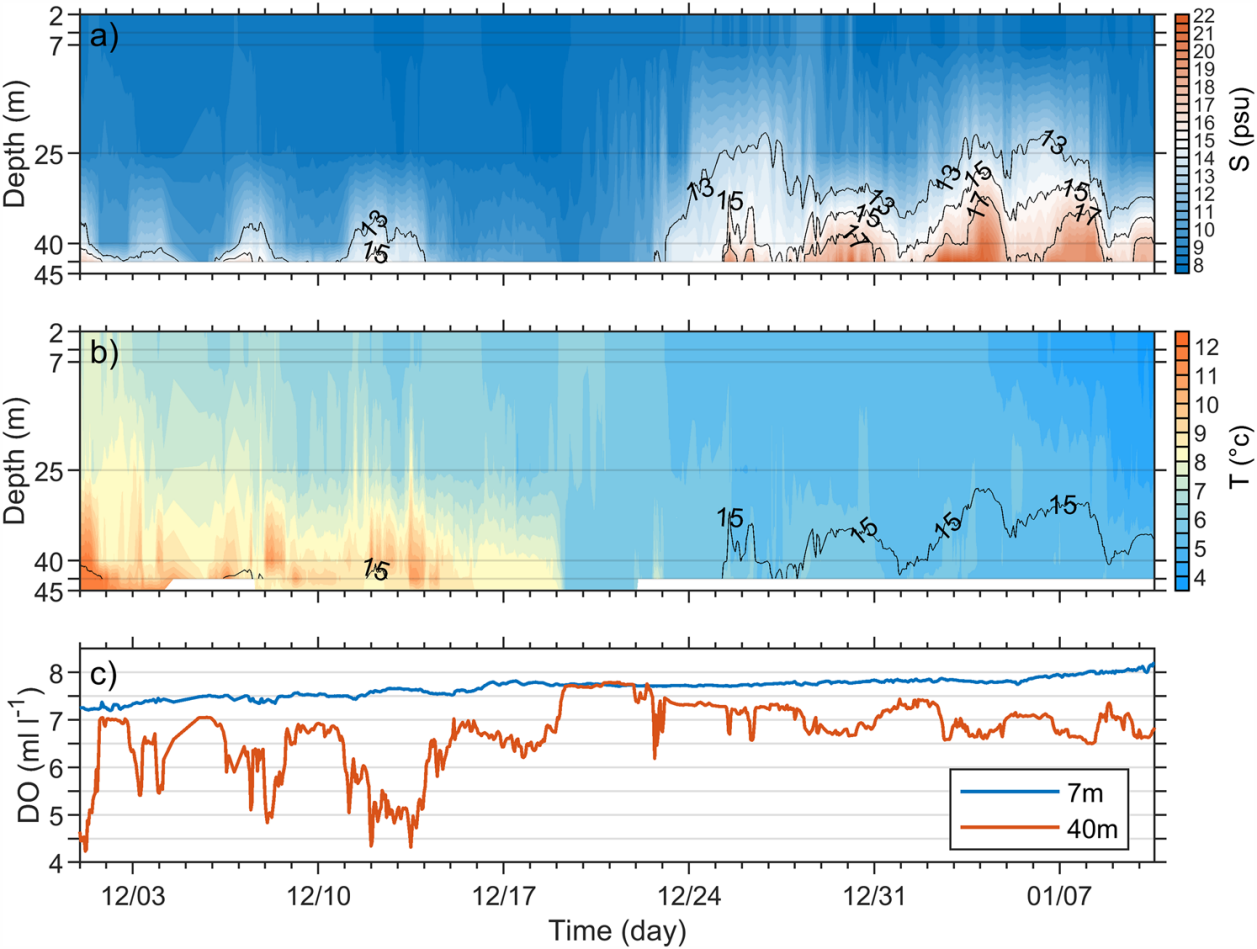
Vertical profiles of salinity (**a**) and temperature (**b**) from 1 December 2023 to 10 January 2024 at the Arkona Basin bouy (ARK). The dissolved oxygen concentrations at 7 m and 40 m are depicted in (**c**). The isohalines of 13, 15, and 17 psu are given in the salinity time series in (**a**) and the isohaline of 15 psu is shown again in (**b**). Hourly averages of the original higher resolution measurements were used here. The vertical axes in (**a**) and (**b**) not only indicate the depth, but also show the position of the instruments in the water column. Salinity at 45 m was not available due to a lack of measurements.

Figure 5 illustrates the salinity, temperature and dissolved oxygen concentration at the Arkona Basin buoy (ARK) from December 2023 to January 2024. On 23 December 2023, the saline inflow reached the ARK and increased the water salinity to 13 psu. Two days later, on 25 December, the bottom salinity exceeded 15 psu. In contrast to conditions at the DAR, the bottom water salinity at ARK increased to values higher than 17 psu with maxima of 19.5, 21.3 and 22 psu on 25 December 2023, 30 December 2023 and 2 January 2024 respectively. The isohaline of 13 psu ascended to 20 m water depth (Fig. 5a). The occurrence of such high salinities at ARK indicates that the inflow through the Sound reached this station, since salinities at DAR were lower throughout the inflow period. Along its way, the inflowing salty water certainly entrained surrounding brackish water, which resulted in a downstream decrease in salinity. As the maximum salinity recorded at ARK was 22 psu, we assume that the salinity in the Sound Sea was well above this value during the event.

The development of the vertical temperature distribution at the ARK is shown in Fig. 5b. On 19 December 2023, due to turbulent exchange processes with the atmosphere the bottom water temperature decreased from 8  to 5.9 °C, a sudden drop of more than 2.1 °C in a few hours . The inflowing water arriving on 22 December had a temperature of about 5.7 °C and temperatures remained below 6 °C throughout the event. The cold water was also rich in oxygen, increasing the dissolved oxygen concentration at 40 m from 6.7 to 7.7 $\mathrm {ml\,l^{-1}}$ by 18 December 2023 (Fig. 5c).

### Calculation of volume, salt and oxygen transport

Applying the SLH differences of about 57 cm at Landsort Norra within 14 days between 15 and 29 December 2023 in Eq. (1), the total volume change in the entire Baltic Sea was estimated to amount about 198 $\mathrm {km^{3}}$. For comparison, the mean climatological annual freshwater surplus (sum of river run-off, precipitation, and evaporation) in the Baltic Sea is about 480 $\mathrm {km^{3} a^{-1}}$^28,29^. The total volume change can be separated between the Belt Sea and the Sound Sea (see Methods) and then amounts to 169 and 29 $\mathrm {km^{3}}$, respectively, giving a ratio of 8:1.4, which is close to the previously estimated ratio of 8:2^30^. The recent volume change is comparable to the volume transport induced by the storms Christian in October 2013 (201 $\mathrm {km^3}$^11^) and Ev and Feliz in March 2014 (203 $\mathrm {km^3}$^12^) and significantly larger than the volume change induced by the storm Xaver in December 2013 (147 $\mathrm {km^3}$^11,12^). It has been shown that the consecutive events in late 2013 (storm Christian) and early 2014 (Stroms Ev and Feliz), without being listed as strong MBIs, had an effective ventilation impact in the deep Gotland basin^12^.

However, the total volume change cannot directly account for changes in the hydrographic properties, which are linked to the actual flow of dense and salty water. Thus, the main inflow period was characterised as the time from the arrival of the highly saline water at DAR (21 December 2023 17:00) and the maximum of the SLH in the Baltic Sea (29 December 2023 17:00)^19^. Hence, a total volume of 75 $\mathrm {km^{3}}$ of highly saline water entered the Baltic Sea. This was distributed between the Belt Sea and the Sound Sea with 62 $\mathrm {km^{3}}$ and 13 $\mathrm {km^{3}}$ respectively. To estimate the salinity of the inflowing water, we analysed salinity data from the Fehmarn Belt Buoy (FEB), which only covers a short period (a few days), as shown in Supplementary Fig. 1. The salinity of the inflowing water in the FEB varied between 21.5 and 23.3 psu. Considering the maximum salinity of 23.3 psu at the FEB and 16.98 psu at the DAR, there was a decrease of 6.3 psu within 115 km distance between the stations, i.e. a dilution rate of about 5.5$\times$ $10 ^{-2}$ psu $\hbox {km}^{-1}$ along this pathway. Due to the lack of data, the dilution rate at the Drogdon Sill cannot be estimated directly and our best possible approach was to apply the dilution rate from FEB to DAR here as well . Considering the distance of 100 km between the Drogdon Sill and the ARK, the salinity of the inflow event can thus be assumed to be 28 psu in the Sound, which is close to measurements of earlier events^21^. The total average salt transport into the western Baltic Sea was estimated with 1.75 Gt (1.39 Gt via the Belt Sea and 0.36 Gt via the Sound). A similar salt import of 1.5–1.7 Gt was observed for a medium range MBI due to a relatively strong westerly wind in November 1996^20,31^. Using the mean dissolved oxygen concentration of 7.5 $\mathrm {ml\,l^{-1}}$ during the inflow event and the total saline inflow volume of 75 $\mathrm {km^3}$, the total amount of oxygen transported to the Baltic Sea was estimated to be 0.8 $\mathrm {10^6}$ t. This corresponds to 40% of the total amount of oxygen transported within the December 2014 MBI^26^. Our transport estimates can of course only be rough assessments, due to the lack of oceanic data at key points and the assumptions made for the calculations. However, these calculations help to integrate the December 2023 MBI into the series of known events and to contextualize its properties and possible impacts.

## Discussion

The time needed for the advection of high saline inflowing water from the Kattegat into the western Baltic Sea depends on the route it takes. For the shorter route through the Sound Sea (about 100 km), it was reported to take about 1–3 days, while the longer route through the Great Belt, Fehmarn Belt, and Mecklenburg Bight (about 300 km) was determined to take 1–2 weeks^19^. The comparison of salinity and arrival time of inflowing water at DAR and ARK stations following storm Zoltan revealed interesting features that contradicted these findings from previous studies, where water from the Sound reached the Arkona Basin before the arrival of flow through the Belt Sea^26^. The tongue of the inflowing water with ($S>$ 13 psu) reached the ARK with a time delay of about 2.9 days compared to DAR. For the presence of the core of the inflowing water ($S>$ 15 psu), the time lag between ARK and DAR further increased to 3.4 days.

Measurements at the FEB, (Supplementary Fig. 1) showed that the salinity of the inflowing water varied between 22 and 23 psu and was diluted at a rate of 5.5 psu per 100 km along its pathway at the Darss Sill (115 km), and arrived at the DAR with a mean salinity of 16 psu. Salinity data was not available for the passage through the Sound. If we assume a similar dilution rate along this pathway as was calculated for Darss Sill, the salinity of the Sound Sea can be estimated to have been about 27–28 psu. Using these values for salinity the salt transport during the inflow event in December 2023 can be estimated to have been between 1.7 and 1.8 Gt.

Despite the lower salinity observed at the DAR, the salt transport through the Belt Sea (1.36 Gt) was significantly higher than that from the Sound Sea (0.35 Gt) with a ratio of about 8:2. Regarding the uncertainty of the estimate, the calculation of the salt transport into the western Baltic Sea in this event exhibits a sensitivity of up to 4.5% to a 1-psu salt variation at the inlets, with 4% attributed to the Belt Sea and 0.5% to the Sound Sea. Using the intensity index $\mathrm {Q_{FM96}}$^19^, the inflow of December 2023 was classified as a moderate inflow ($\mathrm {Q_{FM96}}$ = 17.5). Compared to previous events, the intensity of the December 2023 event was similar to the event in January 2003^25,26^. This was, however, the strongest MBI in the Baltic Sea since 2016 ( Supplementary Fig. 2). Since only very strong MBIs are able to ventilate the central basin of the Baltic Sea^23,26,33,34^, it is likely that storm Zoltan, alone, despite its high wind speeds, did not bring enough saline water into the western Baltic Sea to reach the central basins. Earlier observations during the stagnation period from the beginning of 1995 to the end of 1996 in the deep waters of the Gotland and Fårö Deeps showed that a medium-range MBI in November 1996 did not ventilate these basins significantly. The stagnation period was only interrupted from February to May 1997 due to a series of small inflows.^35^. It is thus essential to note the significance of smaller events, particularly those occurring just before and after an MBI event, which are important for the central deep basin ventilation. This has been demonstrated through the comparison of MBIs in 2003 and 2014, where the earlier event, despite its weaker strength, had a greater impact on the ventilation in the Gotland Basin^8^. Whether the event of December 2023 will contribute to the ventilation of the central basins remains unknown yet and will need to be verified by observations and numerical simulations in the future.

## Methods

Time series of wind speed and direction, ocean currents, temperature, salinity, and dissolved oxygen were collected from three permanent autonomous stations (FEB, DAR and ARK) of the German Marine Monitoring Network (MARNET, Fig. 1). Two stations (DAR and ARK) were operated and maintained by the Leibniz Institute for Baltic Sea Research Warnemünde (IOW) on behalf of the Federal Maritime and Hydrographic Agency (BSH). The FEB is directly maintained by the BSH itself. Each station was equipped with temperature, conductivity (salinity), and dissolved oxygen sensors at different vertical levels depending on the water depth. At the FEB, temperature and salinity measurements were obtained at five depth levels: 3, 6, 15, 20, and 24 m using SeaBird SBE-SMP-ODO instruments. At 3 m water depth no data was recorded. Dissolved oxygen was measured at depths of 6 m and 24 m. At the DAR, temperature and salinity measurements were obtained at seven depth levels: 2, 5, 7, 12, 17, 19, and 21 m using SeaBird SBE 37-IMP-ODO instruments. Additionally, dissolved oxygen was measured at depths of 7 m and 19 m using an optical sensor of SeaBird SBE 63 which was integrated into the measuring system. At the ARK, similar thermosalinometers were used at eight depth levels: 2, 5, 7, 16, 25, 33, 40, and 43 m. Dissolved oxygen data were obtained at water depths of 7 and 40 m with similar sensors. The temporal resolution of the data was 10 minutes, and it was averaged to hourly mean values. Following the manufacturer, the accuracy of a single measurement was 0.002 °C for temperature and 0.003 $\mathrm {mS\,cm^{-1}}$ for conductivity, the depth accuracy was 0.1% of full scale range, and the dissolved oxygen accuracy was 0.07 $\mathrm {ml\,l^{-1}}$. Because of sensor malfunctions, no data was recorded at the deepest level at the DAR and ARK. An upward looking bottom mounted Acoustic Wave and Current Profiler (AWAC) recorded the ocean currents at the DAR. The data were collected in an hourly interval with a bin size of 1 m and with an accuracy of ± 1% of measured values. The quality controlled data were presented here. In addition, each station registered wind speed and direction at 10 m above sea level. The data sets are freely available in hourly resolution at https://insitu.bsh.de. The global wind and gust data used in this study were obtained from the hourly reanalysis dataset of ERA5 provided by ECMWF^32^. In addition, we used the sea level height (SLH) from a tide gauge station at Landsort Norra, which is located to the south of Stockholm (Fig. 1). The Landsort Norra station is very close to the node lines of seiches in the Baltic Sea^36,37^, meaning that it is the best representation of the filling state of the central Baltic Sea^9,20,26,38^. The SLH data were low pass filtered with a cut off frequency of 3 days to remove variations with periods of less than a day. The tide gauge data at Viken and Klagshamn at the north and south of the Sound Sea were used to estimate the inflowing volume through the Sound Sea. The tide gauge data were obtained from the online database of the Swedish Meteorological and Hydrological Institute (https://www.smhi.se/data/oceanografi/).

The volume change in the Baltic Sea ($\mathrm {\Delta V_{BS}}$) was estimated using Eq. (1), where the change of volume is in $\mathrm {km^3}$, $\eta$ is the SLH change at Landsort Norra in cm, $\mathrm {\Delta t}$ is the time period of SLH change in days^9,39,40^. The daily freshwater run off was considered as 1.3 $\mathrm {km^3 d^{-1}}$.

1 $$\Delta V_{{BS}} = 3.8\;{\text{km}}^{3} \;{\text{cm}}^{{ - 1}} \times \Delta \eta - 1.3\;{\text{km}}^{3} \;{\text{d}}^{{ - 1}} \times \Delta t$$

The difference between the total volume and the volume through the Sound Sea can then be used to estimate the volume inflowing through the Belt Sea.

The intensity of the inflow was assessed using the parameter $Q_{FM96}$^19^ which is based on the total amount of salt (M) in giga tons ($\mathrm {10^{12}\,kg}$) transported through the Belt Sea ($\mathrm {M_B}$) and the Sound ($\mathrm {M_S}$) into the Baltic Sea during the inflow event. $\mathrm {M_B}$ and $\mathrm {M_S}$ are the salt transports through the Belt and Sound Seas and are derived from the salinity at the Fehmarn Belt and Drogden Sill and the volume change through these channels. To make the intensity index unitless, the mass of salt is divided by $10^{11}$ kg.

2 $$\begin{aligned} Q_{FM96}= \frac{M_B+M_S}{0.1\,\text{Gt}} \end{aligned}$$

## Summary and conclusion

Observations from different stations were used to study the chracteristics and impacts of the inflow event into the western Baltic Sea from December 2023 to January 2024. A persistently strong westerly wind occurred during the passage of a low-pressure system, storm Zoltan, from 15 to 29 December 2023, and an MBI was induced. The December MBI inflow increased the SLH of the central Baltic Sea by +57 cm in 14 days. The total volume change was about 198 $\mathrm {km^{3}}$, with 75 $\mathrm {km^{3}}$ of highly saline water, which transported a total amount of about 1.7–1.8 Gt salt and 0.8 $\mathrm {10^6\,t}$ oxygen into the Baltic Sea. The inflow was classified to be of moderate intensity and was the strongest inflow since 2016. Further research and hydrographic cruises are needed to understand the effects of the inflow on the deep basins in the central Baltic Sea.

### Supplementary Information

Supplementary Information.

## Data Availability

All data from measuring station (Temperature, Salinity, Oxygen, current characteristics) and large scale wind data are online available at https://insitu.bsh.de, https://cds.climate.copernicus.eu/. The Sea Level Height data are available online on https://www.smhi.se/data/oceanografi/ladda-ner-oceanografiska-observationer#param=sealevelrh2000,stations=core,stationid=2507https://www.smhi.se/data/oceanografi/ladda-ner-oceanografiska-observationer#param=sealevelrh2000,stations=core,stationid=2228https://www.smhi.se/data/oceanografi/ladda-ner-oceanografiska-observationer#param=sealevelrh2000,stations=core,stationid=2095
