# Supplementary material for: Observation of a moderate major Baltic Sea inflow in December 2023
Source: Sci Rep. 2024 Jul 17;14:16577. doi: 10.1038/s41598-024-67328-8 (PMC11254922; doi:10.1038/s41598-024-67328-8)
Supplement: Supplementary file 1 — Supplementary Information. [file 41598_2024_67328_MOESM1_ESM.pdf]

Observation of a moderate Major Baltic Sea inflow in December 2023

Kaveh Purkiani[1], Kerstin Jochumsen[1], Jens-Georg Fischer[1]

[1] Federal Maritime and Hydrographic Agency of Germany, Hamburg, Germany

Supplementary section:

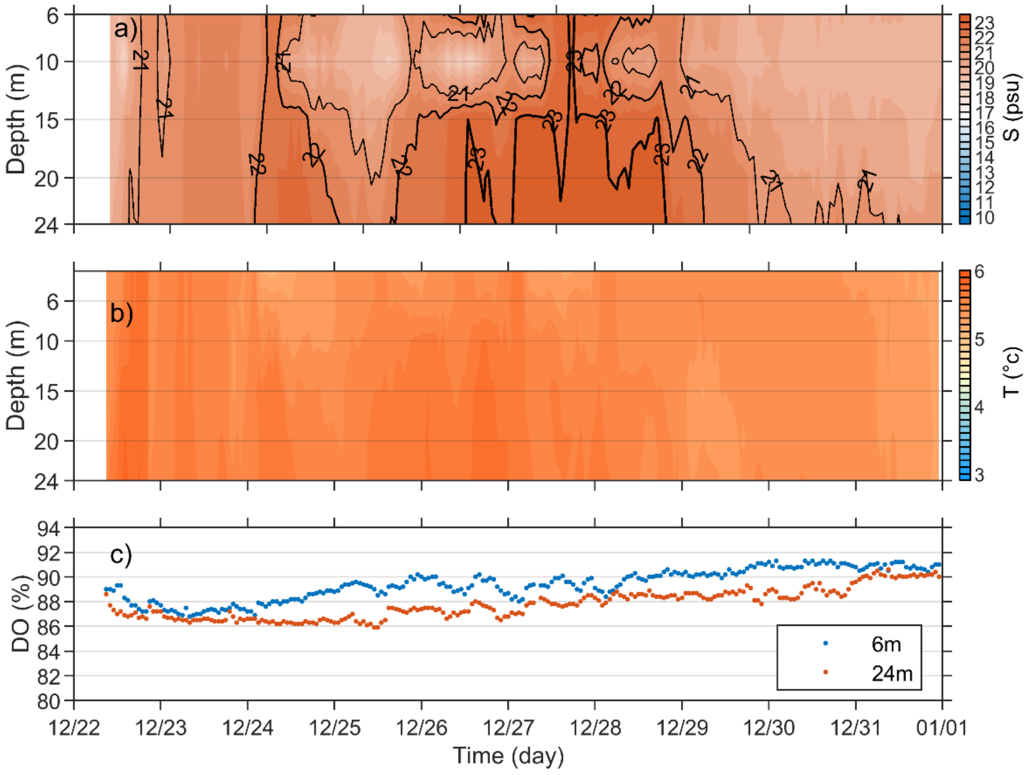

Supplementary figure 1: Vertical profile of (a) salinity and (b) temperature from 22 December 2023 to 1 January 2024 at the Fehmarn Belt station (FEB). The dissolved oxygen concentration at 6m and 24 m are depicted in (c). The isohalines of 21, 22, and 23 psu are given in the salinity profile. The vertical axis in (a) and (b) not only indicate the depth, but also show the position of the measuring sensors. Hourly averages of the original higher resolution measurements are shown here.

Water salinity in the Fehmarn Belt (FEB) was between 21 and 23.3 psu in the whole water column from 22 December to 30 December 2023, when the main inflow passes through (supplementary figure 1a). The main inflow had a temperature of 5.5-6°C (supplementary figure 1b). The dissolved oxygen concentration of bottom water at 24 m reached to the values of surface (> 88%) during the passage of the inflow (supplementary figure 1c).

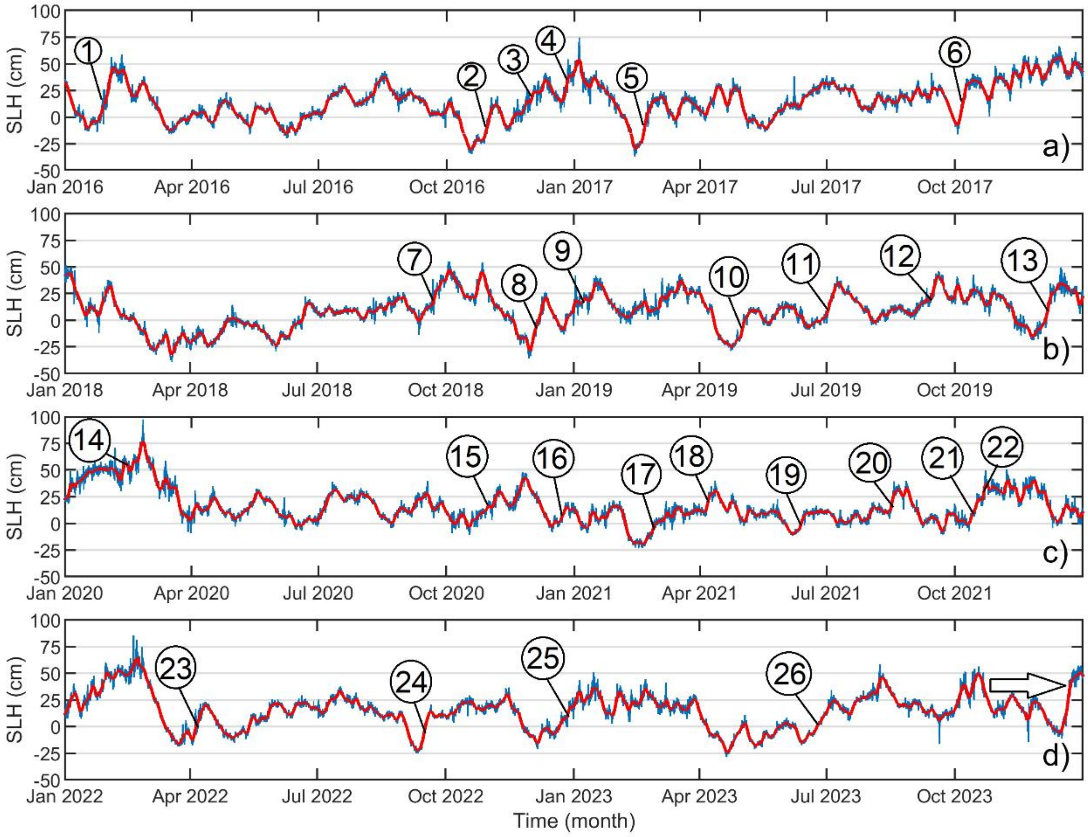

Supplementary figure 2: Hourly sea level change (blue line) at the Landsort Norra from 2016 to 2023. The red thick line depicts the smoothed data using a moving average data filter with a cutoff period of 3 days. MBIs are detected and depicted as numbers. The arrow shows the recent event of Zoltan and its relevant sea level height. 8-year data segmented into 4 subplots (a) to (d), each covering 2 years for concise presentation.

We have analysed the long-term sea level height (SLH) data from 2016-2023 and the results are shown in supplementary figure 2. To better illustrate the data, each panel shows 2 years of SLH variability, i.e. (a) 2016-2017, (b) 2018-2019, (c) 2020-2021, and (d) 2022-2023, where about 27 inflow events with different volume transports were detected. The December 2023 MBI (shown as arrow with ID27) stands behind the January-February 2016 MBI (ID1) with 243 km^3, which imports 2.5 Gt of salt into the Baltic Sea. Applying the SLH differences of each event in equation (1), the total volume change of every event was calculated. Supplementary table 1 summarizes the results. The events marked in supplementary table 1 have a salt transport greater than 1 Gt. Among them, except for the MBI 2016 (ID1, 2.5 Gt), the recent event of Zoltan (ID27) had a salt transport larger than 1.7 Gt.

Event ID	SLH Variation (cm)	Volume Transport (km^3)
1	56	240
2	43	181
3	50	170
4	34	179
5	51	210
6	42	184
7	48	230
8	52	212
9	22	120
10	32	101
11	35	130
12	32	116
13	50	160
14	35	112
15	32	108
16	16	52
17	34	90
18	20	68
19	23	62
20	22	70
21	23	75
22	18	72
23	33	125
24	37	140
25	46	145
26	42	138
27	57	198

Supplementary table1: ID number, sea level height, and volume transport of each MBI depicted in supplementary figure 2.
